# What’s the situation with ocular inflammation? A cross-seasonal investigation of proteomic changes in ocular allergy sufferers’ tears in Victoria, Australia

**DOI:** 10.3389/fimmu.2024.1386344

**Published:** 2024-05-24

**Authors:** Esrin Aydin, Shuai Nie, Serap Azizoglu, Luke Chong, Moneisha Gokhale, Cenk Suphioglu

**Affiliations:** ^1^ NeuroAllergy Research Laboratory (NARL), School of Life and Environmental Sciences, Deakin University, Waurn Ponds, VIC, Australia; ^2^ School of Medicine (Optometry), Deakin University, Waurn Ponds, VIC, Australia; ^3^ Bio21 Mass Spectrometry and Proteomics Facility, Bio21 Molecular Science & Biotechnology Institute, The University of Melbourne, Melbourne, VIC, Australia

**Keywords:** ocular allergy, proteomics, inflammation, allergic rhinoconjunctivitis, angiogenesis, wound healing, protein biomarker, human tears

## Abstract

**Background:**

Ocular allergy (OA) is a localized subset of allergy characterized by ocular surface itchiness, redness and inflammation. Inflammation and eye-rubbing, due to allergy-associated itch, are common in OA sufferers and may trigger changes to the ocular surface biochemistry. The primary aim of this study is to assess the differences in the human tear proteome between OA sufferers and Healthy Controls (HCs) across peak allergy season and off-peak season in Victoria, Australia.

**Methods:**

19 participants (14 OA sufferers, 5 HCs) aged 18–45 were recruited for this study. Participants were grouped based on allergy symptom assessment questionnaire scoring. Proteins were extracted from human tear samples and were run on an Orbitrap Mass Spectrometer. Peaks were matched to a DIA library. Data was analyzed using the software MaxQuant, Perseus and IBM SPSS.

**Results:**

1267 proteins were identified in tear samples of OA sufferers and HCs. 23 proteins were differentially expressed between peak allergy season OA suffers vs HCs, and 21 were differentially expressed in off-peak season. Decreased proteins in OA sufferers related to cell structure regulation, inflammatory regulation and antimicrobial regulation. In both seasons, OA sufferers were shown to have increased expression of proteins relating to inflammation, immune responses and cellular development.

**Conclusion:**

Tear protein identification showed dysregulation of proteins involved in inflammation, immunity and cellular structures. Proteins relating to cellular structure may suggest a possible link between OA-associated itch and the subsequent ocular surface damage via eye-rubbing, while inflammatory and immune protein changes highlight potential diagnostic and therapeutic biomarkers of OA.

## 1 Introduction

Ocular allergy (OA) is a localized form of allergy triggered by commonly occurring airborne allergens such as pollen, animal dander, pollution, dust, and fungal spores (1, 2). Upon contact with the ocular surface, allergic peptides bind to Immunoglobulin E (IgE) receptors on the surface of mast cells, causing a degranulation cascade that results in symptoms of itchiness, inflammation, redness of the ocular surface, and swelling of the eyelids (3). Allergic rhinitis, when combined with symptoms of OA, is commonly referred to as hay fever. Hay fever is a seasonal allergic condition that typically manifests as signs and symptoms of itchiness, inflammation, irritation, runny eyes/nose, wheezing or redness (4). These signs and symptoms vary depending on the individual. Hay fever is highly prevalent in Australia, with a marked increase in prevalence from 15.5% to 23.9% among adults from 2001 to 2022 (5). Victoria has been shown to have the second highest prevalence of hay fever in Australia as indicated by a consistently greater proportion of hay fever sufferers than the Australian average. In 2022, the Victorian prevalence was 34.4%, well above the Australian average of 23.9% (5). The prevalence of hay fever is particularly concerning due to the hallmark OA symptom of itch, as OA sufferers often treat their symptoms with unmonitored use of over the counter medications and seek relief from itchiness via excessive eye-rubbing.

Changes to the ocular surface biochemistry resulting from OA-associated itch, inflammation and eye-rubbing have not yet been investigated in an Australian population. Early findings by Weed et al. have identified a physiological link between an irreversible degenerative corneal ectasia condition called keratoconus and OA (6). In keratoconus, the cornea begins to thin and sag over time, causing progressive vision loss that can be exacerbated by eye-rubbing (7). Excessive eye-rubbing due to OA is thought to be a trigger for this condition due to increased mechanical stress and pressure, potentially causing changes to the shape of the cornea and the ocular surface biochemistry. A 2008 study looking at co-morbidities of keratoconus found a strong correlation between hay fever sufferers experiencing OA symptoms and keratoconus concurrently (p=0.007) (6). This indicates that allergy-associated itch, and thus eye-rubbing, likely has a direct effect on the ocular surface at a tissue and cellular level. Further research into biochemical changes occurring on the ocular surface due to allergy, and the potential for identification of biomarkers, have been the focal point of emerging corneal ectasia research to better understand conditions such as keratoconus. Studies have found differential expression of proteins such as keratins, immunoglobulins and other typical ocular surface proteins related to structural composition, inflammation and homeostasis occurring in keratoconic individuals compared to Healthy Controls (HCs) that may be utilized in future research as biomarkers of keratoconus (8, 9).

Biomarker discovery has played a crucial role in emerging research in a wide variety of ocular (10–13) and non-ocular diseases (14, 15). In recent years, biochemical markers of disease used to accurately identify disease state or level of disease progression have become increasingly important in medical research. These biochemical markers -or biomarkers- can include proteins, lipids, metabolites, genes or any other biochemical change able to be used to distinguish disease states from HCs. OA biomarker research has not yet been studied in sufficient detail but would greatly assist with establishing a strong biochemical link between OA and corneal ectasia for the first time, while also identifying a myriad of new targets for *in vitro* and *in vivo* studies of OA biopathways and treatments. Starting with proteomic studies would be advantageous, as it allows for a broad overview of the tissue-level changes occurring within biological samples such as human tears and can be built upon for a full overview of ocular surface changes occurring in OA.

Li et al. and Tomazic et al., both previously used Mass Spectrometry (MS) techniques of Liquid Chromatography-Mass Spectometry/Mass Spectrometry (LC-MS/MS) and Matrix-Assisted Laser Desorption/Ionization-Time of Flight Mass Spectrometry (MALDI-TOF/TOF) to assess proteomic changes to the ocular surface in human tears of OA sufferers compared to HCs (16, 17). Despite similarities in analytical techniques, their sample collection and preparation techniques were not the same (16, 17). As such, their findings did not show any overlapping significantly differentially expressed proteins despite both being shotgun techniques, indicating that perhaps the combination of sample collection, preparation and analysis methods used were too dissimilar for comparison (18). The most recent study by Neil et al. in 2020 used Automated Electrophoretic Technology (AET) to characterize the proteome of OA sufferers’ tears (19). AET has become more popular in recent years due to its reduced financial cost and run time, but it lacks the sensitivity of MS techniques such as LC-MS/MS (20).

Tomazic et al. also investigated the longitudinal effect of peak allergy season vs off-peak season on the tears in a Letter to the Editor published in 2018 (17). This piece showed that only Beta-2 Microglobulin, Lactotransferrin (LTF) and Lipocalin-1 (LCN1) were significantly differentially expressed between OA and HCs in peak allergy season; and cystatin-S, lactoperoxidase, and zymogen granule protein 16 homolog B were differentially expressed in off-peak season (17). This ultimately demonstrated that there were differences occurring in the ocular surface proteome between OA sufferers and HCs even during off-peak seasons, but lacked sufficient sensitivity as evidenced by detection of only 90 total proteins, of which only 22 were assessed for statistically significant differential expression after data filtering. Additionally, the Tomazic study took place in Austria, where the climate during off-peak season is vastly different to that of Australia. In Australia, during the off-peak season, allergens such as grass pollens are still airborne (21) and thus able to initiate allergic responses, albeit at to a lesser extent. This may have a large impact on expression of homeostatic and protective proteins on the ocular surface in Australian OA sufferers compared to European OA sufferers experiencing colder climates in off-peak season.

This study therefore aims to assess the differential expression of proteins in human tears of OA sufferers versus HCs in both peak allergy season and off-peak season with a previously unmet level of sensitivity in OA studies. The addition of a cross-seasonal component to this exploratory pilot study is highly beneficial for studying potential protein biomarkers of OA across multiple seasons, and is an early step in validating associations found between biomarkers and symptom expression. This research will be instrumental for future applications in OA diagnostics and therapeutics to reduce and investigate the potentially harmful effects of inflammation, irritation and eye-rubbing on the ocular surface of OA sufferers.

## 2 Methods

### 2.1 Study population

This study was conducted in accordance with research ethics guidelines as advised by the Deakin University Human Research Ethics Committee (DUHREC study #2021–189). The study population consisted of 19 adults aged 18–45 from Victoria, Australia. Written informed consent was obtained from all participants prior to study commencement. 14 OA sufferers and 5 HCs were included in the study. Participants attended two sessions each, one during peak allergy season (November 2021, Spring) and one during off-peak season (June 2022, Winter).

### 2.2 Clinical exam sequence

Both sessions began with questionnaires assessing participant demographics, ocular health history, and symptom and Quality of Life (QoL) assessments. Best-corrected vision was assessed using an electronic LogMAR chart, followed by slit lamp assessment of the ocular surface.

Ocular surface slit lamp assessment was carried out before and after tear sample collection. Before tear collection, the ocular surface was inspected for redness using white light and graded using the Efron grading scale (22). Post tear sample collection, participants’ ocular surface was re-assessed using fluorescein dye to check that there was no damage to the ocular surface due to the tests conducted.

### 2.3 Questionnaires

Participants completed the Quality of Life in Children with Vernal Keratoconjunctivitis (QUICK) questionnaire (23), which consists of two sections, one for symptoms score and another for QoL. Both sections are assessed on a scale from 0–6. The symptoms score from this questionnaire was used to classify participants as OA or HCs following conversion to a scale from 0–100 using the following formula:


$$\text{Converted}\;\text{symptom}\;\text{score}=\left(\frac{\text{Total}\;\text{QUICK}\;\text{symptoms}\;\text{score}}{\text{highest}\;\text{possible}\;\text{score}-\text{lowest}\;\text{possible}\;\text{score}}\right))*100$$

A symptom score of greater than 1 was determined to be indicative of OA, as it indicated presence of OA symptoms (24).

The mini Rhinoconjunctivitis Quality of Life Questionnaire (miniRQLQ) was also administered to assess QoL changes due to OA across seasons. An additional demographics questionnaire was used to assess participant eligibility. Participants aged under 18 or over 45 were excluded from the study due to potential age-related bias. Participants were further screened for ocular and general health history, and any recent reporting of ocular infection, surgery or injury was grounds for omission from the study as well as pregnancy and breastfeeding.

### 2.4 Tear sample collection

Tear samples were collected from all participants using the Glass Microcapillary Flow (GMF) method. Approximately 5–45μL of tears were collected from the lateral canthus of each eye and stored on dry ice until transfer to a -80°C freezer. Samples were pooled from both eyes to ensure adequate volume for analysis.

### 2.5 Bicinchoninic acid assay

The Pierce BCA kit (Thermo Fisher Scientific, USA) was used to determine the protein concentration of each tear sample using the microplate protocol outlined in the Pierce BCA protein assay kit handbook. The diluent used for standard and sample preparation was milliQ water. The assay had a detection range of 125–2000μg/mL.

Frozen tear samples were defrosted on ice. A 5μL aliquot of each sample was transferred into labelled sterile Eppendorf tubes. Each aliquot was diluted up to 25μL using milliQ water to prepare for the assay. 10μL of each diluted sample and standard were pipetted into the wells of a 96-well plate in duplicate. 200μL of working reagent (prepared according to the Pierce BCA protein assay kit handbook) was added into each well. The plate was sealed with a plate cover, shaken for 30 seconds at a moderate speed, then incubated for 30 minutes at 37°C. Once it had been incubated, the plate cover was removed, and the plate was run on a Varioskan LUX multimode microplate reader (Thermo Fisher Scientific, USA) at 562nm. A standard curve was generated using SkanIt™ software (Thermo Fisher Scientific, USA) to determine the concentration of each unknown sample in μg/mL.

### 2.6 Protein extraction

Tear samples were diluted to a concentration of 1μg/μL using BCA data and milliQ water. Approx. 11.5μg of protein in 11.5μL milliQ was used per trypsin digestion and S-trap™ column (ProtiFi, USA) purification. 11.5μL of protein was reduced using 0.5μL of 10mM tris (2-carboxyethyl) phosphine (TCEP) at 55°C for 15 minutes. The sample was then alkylated using 2.5μL of 50mM iodoacetamide and incubated in the dark for 45 minutes at room temperature. 2.5μL of phosphoric acid acidifier was then added to a final [v/v] of 2.5%. The sample was diluted 1:6 using a binding and wash buffer consisting of 100mM TEAB in 90% methanol for transfer to the S-trap spin column.

The sample was spun at 4000 x *g* for 30 seconds between each of four total wash steps using 150μL of wash buffer each time. The S-trap was then centrifuged a final time at 4000 x *g* for 1 minute to ensure all buffer was fully removed from the column.

Proteins trapped in the column were digested with 1μg trypsin in 50mM of TEAB. This solution was applied to the top of the column and left to incubate overnight in a 37°C water bath. The next day, peptides were eluted first using 40μL of 50mM TEAB left to sit for 30 minutes at room temperature, then 40μL of 0.2% formic acid, and finally 40μL of 50% acetonitrile [v/v] in distilled water. Each step was followed by centrifugation at 4000 x *g* for 1 minute. The eluent was placed on a Savant SpeedVac™ Vacuum Concentrator (Thermo Fisher Scientific, USA) for 15 minutes at room temperature to remove acetonitrile from the sample. The eluted peptides were then freeze-dried overnight and stored at -80°C until needed for analysis.

### 2.7 Library preparation

The dried peptides were resuspended in 2% acetonitrile/0.05% trifluoracetic acid [v/v] in distilled water to a final peptide concentration of ~0.5μg/μL. 2μL from each sample was aliquoted into a clean, labelled plastic LC column and used for library preparation.

### 2.8 Liquid chromatography- mass spectrometry/mass spectrometry, protein labelling and quantification

Data-Independent Acquisition (DIA) was carried out on the Orbitrap Exploris 480 Mass Spectrometer (Thermo Fisher Scientific, USA). The settings of nano electrospray voltages, ion funnel RF, and capillary temperature were 1.9kV, 50%, 275°C, respectively. A survey scan with a m/z range of 350 to 1400, a resolution of 120,000, a normalized automatic gain control (AGC) target of 250%, a maximum ion trapping time of 50ms was performed before 49 DIA windows with an m/z isolation window of 13.7, a precursor ion m/z range of 361–1033, a MS/MS scan range of m/z 200–2000, a resolution of 30,000, an AGC of 1e6, a maximum ion trapping time of 55ms and a normalized collision energy (NCE) of 30%.

DIA LC-MS data were analyzed with Spectronaut 16 using the direct-DIA default settings. In summary, digestion enzyme specificity was set to trypsin and up to 2 missed cleavages were allowed. Mass tolerance for full scan MS and MS/MS scans were set at dynamic mode, which optimizes the mass tolerance based on pre-search internal mass calibration. Search criteria included carbamidomethylation of cysteine as a fixed modification, as well as oxidation of methionine and acetylation (protein N-terminus) as variable modifications. Data were searched against UniProt human sequence database. The false discovery rate (FDR) was set to 1% at peptide spectrum match (PSM), peptide and protein level. MaxLFQ of the intensity at MS/MS level was enabled for label free quantification and no imputation was enabled. The datasets generated for this study can be found in the ProteomeXchange Consortium via the PRIDE partner repository with the dataset identifier PXD051204.

### 2.9 Statistical analysis

Identification of global protein abundances was executed using MaxQuant Perseus software (v 2.0.9.0). Data were Log_2_ transformed. Two sample t-tests were used to identify differentially expressed proteins using threshold values S0 of 0.1 and was filtered by Log_2_ fold-change (Log_2_FC) of x>1; x<-1 and significance (p<0.05) (9, 25). Data visualization was supplemented using IBM^®^ SPSS^®^ Statistics.

## 3 Results

### 3.1 Participant demographic data

**Table 1** shows the number of participants, gender distribution, mean age, mean QUICK questionnaire symptoms score and mean protein concentration across each of the four groups. No significant differences in gender ratios or age were found between groups (p>0.05).

**Table 1:** Table depicting demographic data for study participants.

*Characteristic*	*Peak allergy season*		*Off-peak season*	
	Ocular Allergy	Control	Ocular Allergy	Control
*Number of participants*	14	5	7	3
*Number of females:males*	10:4	2:3	5:2	2:1
*Age (mean±standard deviation)*	32.71±7.65	28.20±2.17	31.71±8.65	27.67±2.08
*QUICK symptoms score (mean±standard deviation)*	25.83±19.30	0±0	24.40±19.90	0±0
*miniRQLQ score (mean±standard deviation)*	33.84±20.25	9.76±11.52	29.37±18.35	2.38±2.38
*Mean protein concentration (mean±standard deviation) (μg/μL)*	8.12±2.59	7.15±1.73	7.44±1.74	7.17±1.39

No significant differences were observed in age or mean protein concentration between groups (p<0.05). The QUICK symptom score showed a downward trend in allergy symptom scoring ^517^ between peak allergy season and off-peak allergy season in ocular allergy sufferers. QUICK- Quality of Life in Children with Vernal Keratoconjunctivitis Questionnaire; miniRQLQ- mini Rhinoconjunctivitis Quality of Life Questionnaire

Participants from the OA group scored a mean±standard deviation of 27.63±17.31 during peak allergy season and 21.61±16.41 during off-peak allergy season on the QUICK questionnaire, showing a slightly downward trend in symptom severity from peak allergy season to off-peak season.

The mean protein concentration of the tears was 7.6±2.05μg/μL as measured by BCA. No significant differences were found between groups or seasons (p>0.05) for protein concentration.

### 3.2 Most abundant proteins

A total of 1267 proteins were identified in tear samples of OA sufferers and HCs across both seasons using LC-MS/MS. The 10 most abundant proteins among HCs from both seasons are shown in **Table 2**, including lysozyme C (LYZ), LTF, proline-rich protein 1 (OPRPN), immunoglobulin fragments, keratins, phospholipase A2 (PLA2G2A), proline-rich protein 4 (PRR4), and LCN1. None of these proteins showed significant changes in expression between groups during groupwise comparisons.

**Table 2:** Top 10 most abundant proteins in human tears across all participants identified using LC-MS/MS on extracted tear proteins.

*#*	*Gene name*	*Protein name*
**1**	**LYZ**	Lysozyme C
**2**	**LTF**	Lactotransferrin
**3**	**IGHA1**	Immunoglobulin heavy constant alpha 1
**4**	**OPRPN**	Proline-rich protein 1
**5**	**IGHA2**	Immunoglobulin heavy constant alpha 2
**6**	**PLA2G2A**	Phospholipase A2
**7**	**PRR4**	Proline-rich protein 4
**8**	**KRT1**	Keratin, type II cytoskeletal 1
**9**	**LCN1**	Lipocalin-1
**10**	**KRT10**	Keratin, type I cytoskeletal 10

### 3.3 Comparison of the human tear proteome of OA sufferers vs HCs with respect to season

#### 3.3.1 Peak season- OA vs HC

**Figure 1:**
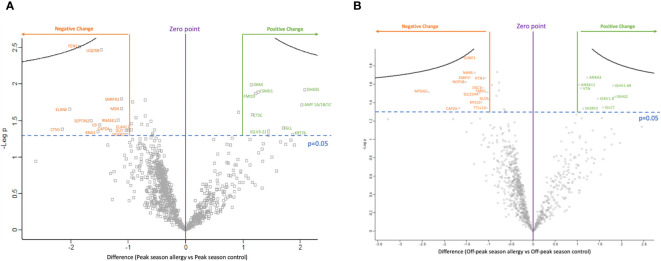
The volcano plots show the significantly differentially expressed proteins between ocular allergy sufferers vs healthy controls after filtering out Log2 fold-changes on the x-axis of >1 or <-1; as well as by significance (p-value<0.05 was considered significant). (A) Volcano plot showing the difference in protein expression between peak allergy season ocular allergy sufferers vs healthy controls. All 14 proteins labelled in orange showed decreased expression in ocular allergy sufferers vs healthy controls during peak allergy season, while 9 proteins labelled in green showed increased expression. (B) Volcano plot showing the difference in protein expression between off-peak season ocular allergy sufferers vs healthy controls. All 13 proteins labelled in orange showed decreased expression in off-peak season between ocular allergy sufferers vs healthy controls, while 8 proteins labelled in green showed increased expression.

Twenty-three proteins showed a significant difference in expression between groups (p<0.05) as shown in **Figure 1A** and in **Supplementary Table S1**. Of these, nine proteins showed increased expression, while fourteen showed decreased expression in OA sufferers vs HCs during peak allergy season.

**Figure 1A** and **Supplementary Figure S1** show all significantly differently expressed proteins in OA sufferers vs HCs during peak allergy season. The top three proteins showing the largest decrease in expression as shown by negative Log_2_FC in OA vs HCs during peak allergy season were cathepsin G (CTSG) (p=0.042), neutrophil elastase (ELANE) (p=0.022), and flap endonuclease 1 (FEN1) (p=0.003). FEN1 also had the most significantly different change in expression between OA and HCs during peak allergy season (p=0.003). The top three proteins with the greatest increase in expression as determined by Log_2_FC were probable ATP-dependent RNA helicase (DHX35) (p=0.012), alpha-amylase (AMY1A/B/C) (p=0.019), and keratin type II cytoskeletal 2 oral (KRT76) (p=0.049).

#### 3.3.2 Off-peak season- OA vs HC

Twenty-one proteins showed a significant difference in expression between groups (p<0.05). Eight proteins showed increased expression among OA vs HC, and thirteen were decreased during off-peak season. An overview of these proteins can be seen in the volcano plot in **Figure 1B** and **Supplementary Figure S1**.

As shown in **Figure 1B** and **Supplementary Figure S1**, the three proteins with the greatest decrease in expression between OA sufferers and HCs in off-peak season as determined by Log_2_FC are myeloid-derived growth factor (MYDGF) (p=0.031), f-actin capping protein subunit alpha (CAPZA1/2) (p=0.046), and Ras GTPase-activating protein-binding protein (G3BP1) (p=0.013). G3BP1 had the greatest overall significance in differential expression between OA sufferers and HCs during off-peak season (p=0.013). The top three proteins with the greatest increase in expression as shown by Log_2_FC include immunoglobulin heavy constant gamma 2 (IGHG2) (p=0.034), immunoglobulin heavy variable 1–69 (IGHV1–69) (p=0.026), and immunoglobulin lambda constant 7 (IGLC7) (p=0.045).

### 3.4 Comparison of longitudinal/seasonal changes in the human tear proteome with respect to allergy status

#### 3.4.1 Seasonal differences among HCs

S1 shows all the significantly differently expressed proteins among HCs, indicating differences in the proteome of control groups during peak allergy and off-peak seasons. Thirteen proteins in total were significantly differentially expressed (p<0.05). Of these, three proteins showed increased expression and nine showed decreased expression in HCs between peak allergy season and off-peak season. Of all the differentially expressed proteins, the three with the greatest degree of negative Log_2_FC are Beta-1,3-galactosyl-O-glycosyl-glycoprotein beta-1,6-N-acetylglucosaminyltransferase 3 (GCNT3) (p=0.031), CUB and sushi domain-containing protein 1 (CSMD1) (p=0.049), and keratin type II (KRT6B) (p=0.001). KRT6B was also the most significantly changed protein among season (p=0.001). HCs between peak allergy season and off-peak season (p=0.001). The top 3 most increased proteins as shown by Log_2_FC are ELANE (p=0.029), IGLC7 (p=0.035), and glutamate receptor-interacting protein 1 (GRIP1) (p=0.039).

#### 3.4.2 Seasonal differences among OA sufferers

Eleven proteins showed significant differences in expression among OA sufferers between peak allergy season and off-peak season (p<0.05). Three were increased in peak allergy season vs off-peak season OA sufferers, while eight were decreased. These changes are shown in S1. The three proteins with the greatest magnitude of decreased expression evidenced by degree of Log_2_FC include keratocan (KERA) (p=0.007), SCAN domain-containing protein 1 (SCAND1) (p=0.039), and transient receptor potential cation channel subfamily M member 3 (TRPM3) (p=0.040). Controversially, the three most increased proteins among OA sufferers between peak and off-peak seasons were probable non-functional immunoglobulin kappa variable 3–7 (IGKV3–7) (p=0.047), serine/threonine-protein kinase 11-interacting protein (STK11IP) (p=0.033), and putative elongation factor 1-delta-like protein (EEF1DP3) (p=0.028). The most significantly differentially expressed protein was keratin type II (p=0.005).

## 4 Discussion

**Figure 2:**
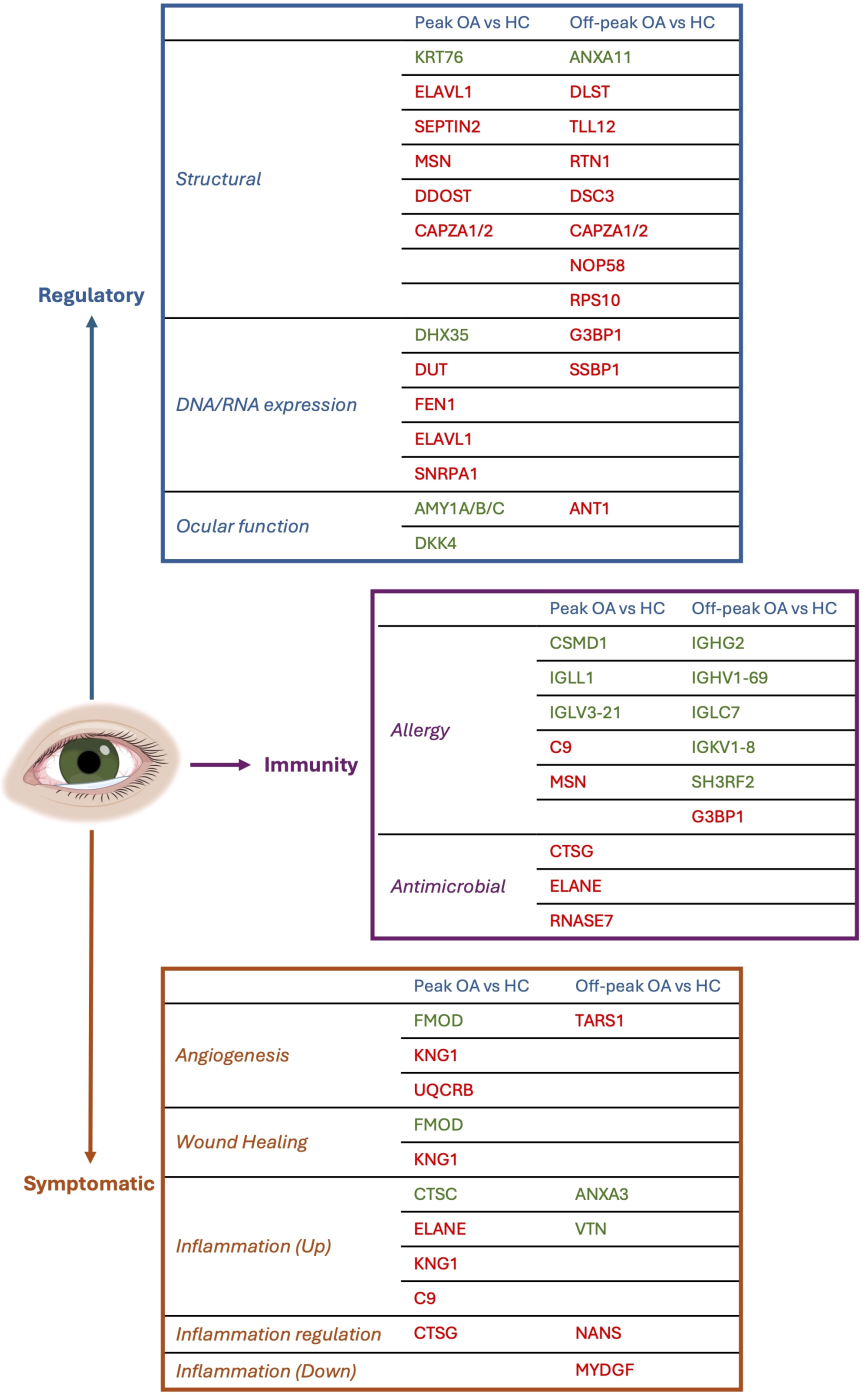
Summary of functions of each differentially expressed human tear protein between Ocular Allergy (OA) sufferers and Healthy Controls (HCs) across both seasons as determined by students t-test. Green protein ID’s are upregulated, while red are downregulated.

The central aim of this study was to assess proteomic biomarkers of OA in human tear samples across peak allergy season (Spring-Summer) and off-peak season (Winter) in Victoria, Australia. Aberrant expression of 23 proteins were detected between OA sufferers and HCs during peak allergy season (**Figure 1A**), and 21 were differentially expressed between OA sufferers and HCs during off-peak season (**Figure 1B**). When compared to one another, only one key cellular structure protein (ie. CAPZA1/2) was consistently downregulated between OA sufferers and HCs across both seasons. Dysregulated proteins were largely linked to immune responses, inflammation, angiogenesis, wound healing and homeostasis across both seasons, indicating potentially damaging changes to the ocular surface of OA sufferers vs HCs, irrespective of season (**Figure 2**).

The healthy tear proteome was comprehensively investigated in a study by Dor et al. in 2019. This study used pooled tear samples collected using Schirmer strips and MS for analysis. Dor et al. showed that in the tears of HCs, proteins such as Immunoglobulin Heavy Constant Alpha 1 (IGHA1), Immunoglobulin Heavy Constant Alpha 2 (IGHA2), LYZ, LTF, PRR4, LCN1 and PLA2G2A were all highly expressed (26). A key limitation of this study was the exclusion of keratins from the study data in order to discuss more functionally linked proteins, however this decision minimizes the role of keratins in maintenance and cell structure on the ocular surface (26). As such, the proteins identified in our study, with the exception of OPRPN, Keratin type I cytoskeletal 10 (KRT10) and Keratin type II cytoskeletal 1 (KRT1), aligned with those of the typical human tear proteome.

The top 10 most abundant human tear proteins consistently expressed across all groups (OA and HCs) are shown in **Table 2**. This study found the most abundant tear proteins to be LYZ, LTF and IGHA1. The high levels of expression of proteins (**Table 2**) are all within expectation, as supported by the literature. Collectively, the findings reported in **Table 2** depict a high level of immunomodulatory and homeostatic regulation occurring on the ocular surface of all participants within this study, irrespective of allergy status. High abundance of OPRPN and PRR4 in the tears indicate stability and moderation of ocular surface functions (27–29) when expressed in conjunction with LYZ, LTF, IGHA1, LCN1 and PLA2G2A. In the tears, these proteins work together to form a network responsible for maintenance of healthy, functioning tear film able to clear airborne microbes and pathogens (19, 29–31). This allows for reduced incidence of infections while balancing lipid-protein-metabolite binding for a variety of tear film maintenance functions on the ocular surface (24).

To understand the baseline profile of healthy tears, expression of highly abundant proteins was investigated in HCs. Differential expression of proteins among HCs between peak allergy season and off-peak season were compared in S1. Interestingly, an overall trend of decreased mucin production, inflammatory regulation, RNA stability and cell metabolism (32–36) can be seen through altered expression of proteins between peak allergy and off-peak seasons. This may be due to weather becoming colder and dryer in off-peak season, thereby necessitating increased mucin production and anti-inflammatory proteins among HCs.

### 4.1 Differentially expressed proteins between OA vs HCs

During peak allergy season, periods of exposure to seasonally linked allergens, such as grass pollen and fungal spores, are more frequent and last longer than during the off-peak season (37). Amidst an allergy flare-up on the ocular surface, an immune response is triggered by re-exposure to allergic peptides, thereby eliciting a symptomatic pathway of inflammation, redness and irritation, followed by return to homeostasis (24). The findings of this study were novel due to the significance of identifying additional key steps in this pathway, theorized to be upregulated during an ocular allergy flare-up. Namely, wound healing and angiogenesis were upregulated in the proteome of OA sufferers’ tears compared to HC for the first time, potentially as a result of allergy-induced itch and eye-rubbing.

Among OA sufferers vs HC during peak allergy season, an expected increase in immune activity was indicated by differential expression of proteins, such as Immunoglobulin lambda-like polypeptide 1 (IGLL1) (Log_2_FC= +1.7), Immunoglobulin lambda variable 3–21 (IGLV3–21) (Log_2_FC= +1.45), CSMD1 (Log_2_FC= +1.26), Complement component C9 (C9) (Log_2_FC= -1.50) and Moesin (MSN) (Log_2_FC= -1.11). These proteins are primarily associated with T and B cell function (38, 39), antigen recognition (40) and complement pathway regulation (41, 42). CSMD1 and C9 may be further linked through immune function, as CSMD1 has been shown to inhibit the complement pathway, thereby leading to downregulation of C9 (42). In allergy, complement pathway activation has been shown to have an immunoregulatory effect via the release of inflammatory and immune-regulating cytokines and chemokines (43). Inhibition of the complement pathway is thereby theorized to affect the differential expression of other immune proteins in OA vs HC during peak allergy season, however this effect was not shown in the off-peak season. Controversially, in the off-peak season, expression of immune proteins IGHG2 (Log_2_FC= +1.84), IGHV1–69 (Log_2_FC= +1.81), IGLC7 (Log_2_FC= +1.57), Immunoglobulin kappa variable 1–8 (IGKV1–8) (Log_2_FC= +1.47) and E3 ubiquitin-protein ligase (SH3RF) (Log_2_FC= +1.13) was significantly increased in OA sufferers vs HC. This indicates that despite the change from peak allergy season to off-peak season, ocular surface changes, such as inflammation, redness and itch are still occurring as suggested by increased immune activity.

Inflammation on the ocular surface has been shown in this study to be modified in OA vs HC. As shown in **Figure 2**, inflammatory proteins such as CSMD1 and Dipeptidyl Peptidase 1 (CTSC) (Log_2_FC= +1.16) were upregulated in OA vs HC during peak allergy season. CSMD1 has been shown to have an anti-inflammatory role (43), suggesting that a net decrease in inflammation was occurring between OA and HC during peak allergy season. This decrease was further highlighted by downregulated expression of C9 and ELANE (Log_2_FC= -2.01). However, concurrent increased expression of Dickkopf-related protein 4 (DKK4) (Log_2_FC= +1.14), a key homeostatic protein (44), may suggest that the decrease in inflammation is indicative of a return to homeostasis on the ocular surface following an allergic flare-up. DKK4 has been shown previously to be primarily expressed in the meibomian gland, which is a secretory gland responsible for maintaining the lipid layer of the ocular surface for antimicrobial and homeostatic maintenance of the ocular surface (44). A prior study using cell-based assays showed that DKK4 directly affected Wnt signaling pathways by inhibiting Wnt gene expression (44). The Wnt cascade is a cell regulation and maintenance biopathway, thereby affecting homeostasis in adult ocular surfaces through reduced meibomian gland function (44). In OA sufferers, this may contribute to irritation and inflammation as the meibomian glands struggle to generate lipids for lubrication of the ocular surface (24) in the presence of upregulated DKK4 (44).

Furthermore, CTSG (Log_2_FC= -2.14), Fibromodulin (FMOD) (Log_2_FC= +1.22) and Kininogen-1 (KNG1) (Log_2_FC= -1.53) have been suggested in the literature to play a key role in mediating inflammation (45–48). KNG1 has a similar function of inflammatory regulation to FMOD (46, 49), but was significantly downregulated in this study between OA vs HC during peak allergy season. Additionally, CTSG and ELANE had the greatest negative Log_2_FC of the differentially expressed proteins in OA vs HC during peak allergy season; and are both linked to inflammatory regulation and microbial clearance (35, 45, 47). Typically, CTSC activates ELANE and CTSG for immune defense at sites of inflammation (47), but this is not the case in the findings reported by this study of OA. Together, these findings may indicate an overall decreased regulation of inflammation on the ocular surface following an allergic flare-up. Downregulation of CTSG and KNG1 among OA sufferers may therefore indicate a decrease in inflammatory regulation occurring on the ocular surface due to long-term allergen exposure, resulting in sustained periods of inflammation over the duration of the peak allergy season as opposed to individual acute flare-ups.

Long-term inflammation is further indicated by increased expression of Vitronectin (VTN) and Annexin 3 (ANXA3) observed during off-peak season amongst OA sufferers vs HC, with a Log_2_FC of +1.09 and +1.22, respectively. Both proteins have been shown to have proinflammatory functions (50, 51), which aligns with the overall increased expression of immune biomarkers on the ocular surface in OA vs HCSs during off-peak season. Similarly, MYDGF, an anti-inflammatory protein (52), has been shown here to be decreased in OA vs HCs (Log_2_FC= -2.36) during off-peak season. The decrease in regulation coupled with increased expression of inflammatory proteins indicates a net upregulation of inflammation on the ocular surface during off-peak allergy season among allergy sufferers. This is consistent with those from peak allergy season, indicating that OA sufferers experience increased inflammation on the ocular surface irrespective of season.

This finding is further supported by downregulated expression of Sialic Acid Synthase (NANS) (Log_2_FC= -1.32) among OA vs HC during off-peak season. NANS mediates scialic acid synthesis in cells, which in turn blocks expression of cytokines on the ocular surface; thus having a net anti-inflammatory effect (53). In the context of this study, NANS expression has been decreased, indicating a dysregulation of cytokine expression on the ocular surface that may cause increased inflammation (53). This may be related to the decreased expression of TARS1 in off-peak season (Log_2_FC= -1.03), a protein secreted in response to Vascular Endothelial Growth Factor (VEGF) and Tumour Necrosis Factor α (TNF-α), cytokines that stimulate angiogenesis (54). Ultimately, angiogenesis may be reduced on the ocular surface, and inflammation may be increased in OA vs HC in off-peak season due to decreased NANS and TARS1.

In this study, inflammation was shown to be closely linked to angiogenesis and vasodilation, manifesting as redness and inflammation on the ocular surface. Angiogenesis and vasodilation refer to the formation and expansion of new blood vessels occurring during an immune response (47, 55). Typically, angiogenesis occurring at sites of inflammation and irritation increases blood flow to the area, allowing for transport of highly important cell mediators such as cytokines, metabolites and other signaling molecules for functions such as wound healing (47, 55). Proteins responsible for stimulation of angiogenesis therefore often overlap with those responsible for inflammation, as evidenced by the upregulation of FMOD (Log_2_FC= +1.22) identified between OA vs HC in peak allergy season. FMOD works by activating the phosphoinositide 3-kinase (PI3K)/the serine/threonine protein kinase B (Akt) signaling pathway (PI3K/Akt pathway), previously linked to both inflammation and angiogenesis (56).

During periods of inflammation and angiogenesis, triggered by allergen exposure, OA sufferers may be inclined to self-manage the ocular itch by rubbing their eyes. This has been shown in the literature to be problematic (7, 57), however the true extent of biochemical changes on the ocular surface resultant from allergic itch-induced eye-rubbing have not been previously characterized. In this study, proteins indicating corneal changes and wound healing resultant from OA were observed in the human tears of OA sufferers vs HC for the first time. Wound healing is the process of repair occurring on a cellular level due to introduced damage to the ocular surface. In OA, this is seen not only through direct implication of wound healing proteins in this study, but also through significantly increased expression of cellular building blocks and structural proteins, such as keratins. On the ocular surface, wound healing may occur as a result of long-term inflammation, eye-rubbing or allergen exposure. In this study, evidence supporting the significant increase in wound healing pathway activation and cell structure changes on the ocular surface was abundant, as reflected through differential expression of structural proteins AMY1A/B/C, KRT76, and wound healing associated proteins FMOD, DKK4, KNG1, MSN, Septin-2 (SEPTIN2) and CAPZA1/2.

**Figure 3:**
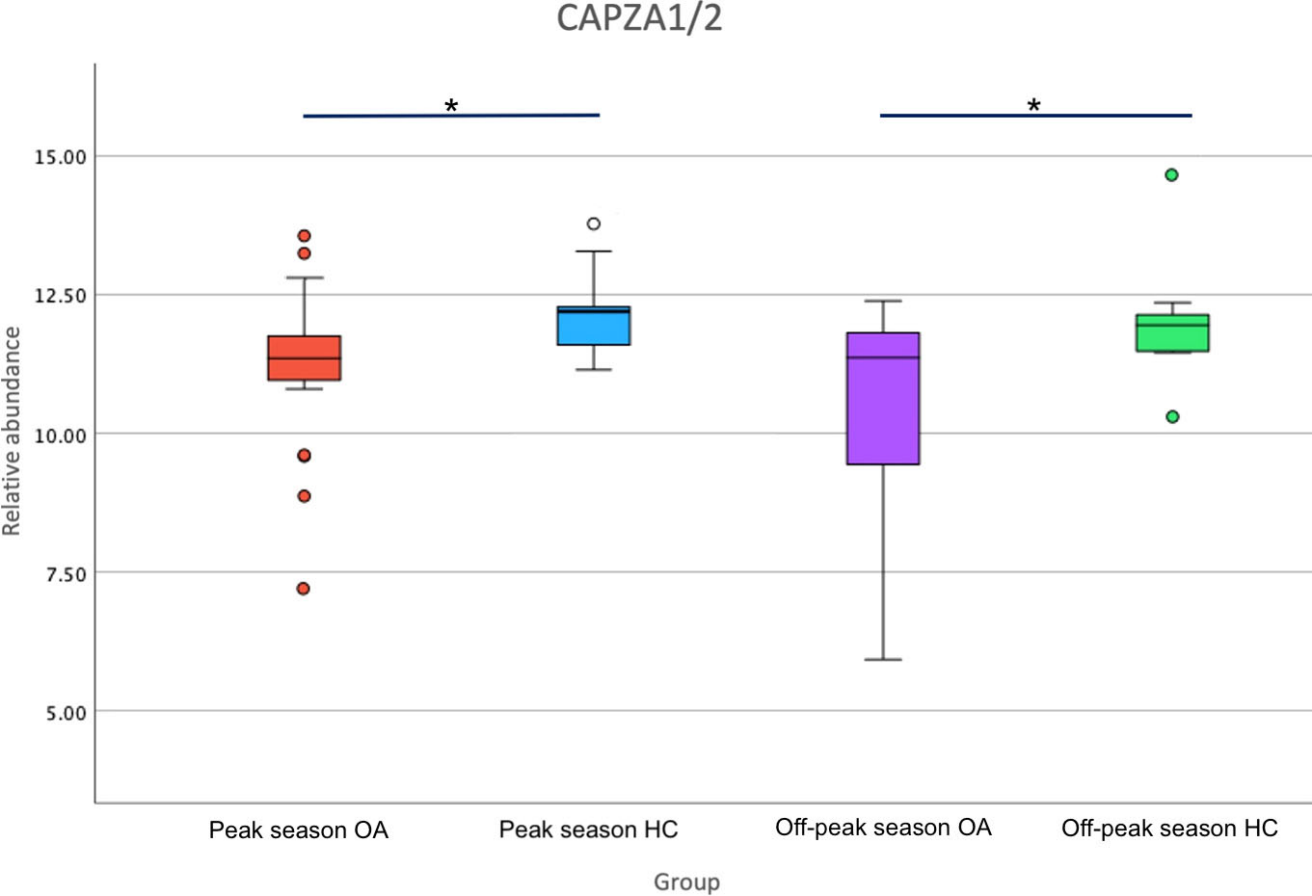
Boxplot of relative abundance of F-actin-capping protein subunit alpha (CAPZA1/2) expression in Ocular Allergy (OA) sufferers vs Healthy Controls (HCs) during peak allergy season and off-peak season. Stars (*) denote significantly different expression (p-value<0.05) between OA vs HCs.

Structural and wound healing proteins such as MSN, SEPTIN2 and CAPZA1/2 were downregulated in tears of OA vs HC during peak allergy season, indicating overall reduced structural protein production, potentially to compensate for allergy-induced eye rubbing (38, 58–60). These structural changes appear be maintained during off-peak season, as evidenced by differentially expressed Annexin 11 (ANXA11), ADP/ATP translocase 1 (ANT1), Tubulin—tyrosine ligase-like protein 12 (TTLL12), Reticulon 1 (RTN1), Desmocollin-3 (DSC3) and CAPZA 1/2. The protein with the second greatest decrease in expression between OA and HCs in off-peak season, as determined by Log_2_FC, is CAPZA 1/2 (Log_2_FC= -1.67), which also showed a significant decrease in expression in peak allergy season (Log_2_FC= -1.03). **Figure 3** showed that the under expression of CAPZA 1/2 in the tears of OA sufferers is conserved between seasons. CAPZA 1/2 has been linked to cell structure regulation and thus may be decreased in OA sufferers due to dysregulation of cellular structure and homeostasis caused by inflammation and allergy-associated itch (58).

**Figure 4:**
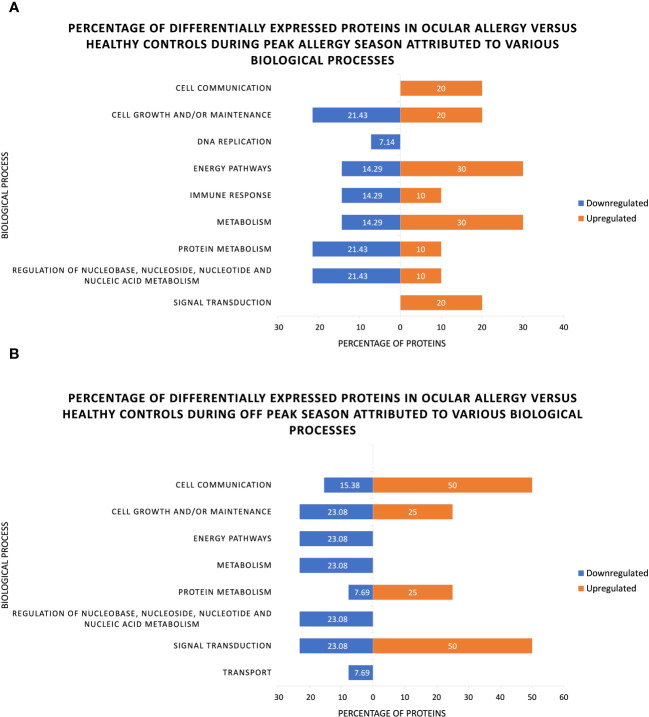
Percentage of significantly up- and downregulated proteins associated with various biological processes as identified through functional analysis of human tear proteins in ocular allergy sufferers and healthy controls during (A) Peak allergy season and (B) Off-peak season.

Return to homeostasis following an allergy flare up is evident when looking at the percentage of differentially expressed proteins pertaining to various biological functions (**Figure 4**). It is thereby evident that the proportion of proteins involved in cell growth/maintenance that were up- or downregulated is almost equal, indicating that the proteins responsible for cell maintenance (and therefore homeostasis) are somewhat balanced on the ocular surface across both seasons.

### 4.2 Discussion of seasonal changes among ocular allergy sufferers

Longitudinal changes occurring among OA sufferers between peak allergy season and off-peak season appear to be largely related to cellular structure and protein synthesis. Keratocan (KERA) (Log_2_FC= -2.53) and Keratin, type II cytoskeletal 1b (KRT77) (Log_2_FC= -1.42), were highly significantly downregulated among OA sufferers across seasons indicating reduced corneal transparency and structure during peak allergy season (8, 9, 61–63). This may be due to increased allergy-induced eye-rubbing and is supported by similarly downregulated expression of TGc domain-containing protein (TGM3) (Log_2_FC= -1.05) and its role in protein formation (64). Additionally, there appears to be dysregulated homeostasis occurring among allergy sufferers between seasons, as indicated by decreased expression of Transient receptor potential cation channel subfamily M member 3 (TRPM3) (Log_2_FC= -1.75) and Basic immunoglobulin-like variable motif-containing protein (BIVM) (Log_2_FC= -1.34). Overall, OA sufferers can be seen to have decreased cell structure, homeostatic capability and lipid regulation in the peak allergy season compared to off-peak (64–68). This indicates general reductions in ocular surface function and therefore poorer ocular health for OA sufferers during peak allergy season.

### 4.3 Limitations

The key aim of this study was to investigate changes to the human tear proteome across multiple seasons among OA sufferers vs HCs. Limitations of this study design therefore largely involved participant retention, collection of tear samples of the required volume from all participants, as well as extracting proteins of sufficient quality for LC-MS/MS analysis. Participants recruited in peak allergy season were asked to return in the off-peak season. Of these, 17.6% dropped out due to scheduling conflicts, illness, COVID-19 exposure/isolation, or were unable to be contacted. Participants with dry eyes, evidenced by low tear volume and slit lamp examination, were dismissed also, reducing participant numbers in the off-peak season further as shown in **Table 1**. Participant tear volume may have been highly dependent on time of day, age and general ocular health/lifestyle factors, such as daily exposure to UV, pollution and screentime as previously shown in the literature (69, 70). Future projects seeking to validate the findings from this study may choose instead to utilize alternative sample collection types, such as impression cytology, that are less subject to individual variations.

Additionally, future iterations of this study would benefit from a significantly increased sample size in order to validate and expand upon findings from this study. Ultimately, due to low sample size, this study was unable to further validate findings by age, sex or other individual characteristics. This study should thereby be taken as an exploratory pilot study of the impact of OA on the human tear proteome over time.

## 5 Conclusion

The key findings of this study indicated that during peak season, there was an overall increase in inflammation, immune activity and corneal structural protein expression; and a decrease in inflammatory regulation and antimicrobial activity. The proteins identified in this study as differentially expressed in OA during peak allergy season or off-peak season have not been previously reported by other comparable studies (18). FMOD and KRT76 were shown to be differentially expressed in a corneal ectasia condition called keratoconus in the literature (8, 61, 63, 71). Converse to findings in the literature regarding keratoconus, FMOD was shown to be increased in OA sufferers during peak allergy season in this study. Both KRT76 and FMOD are suggested by this study to be linked as responses to allergy-induced itch, ultimately increasing structural protein expression, angiogenesis, and wound healing potentially as a result of eye-rubbing on the ocular surface. CAPZA 1/2 was shown to be consistently decreased in OA sufferers compared to HCs irrespective of season. Longitudinal changes to the tear proteome of OA sufferers vs HCs showed minimal difference, with those occurring among the control group, largely occurring in proteins related to cellular structure. Among the allergy groups, seasonal changes identified a mostly consistent expression of proteins.

This study was ultimately successful in its endeavor to identify differences in the human tear proteome of OA sufferers with respect to season within Victoria, Australia, and can be used as a foundation for future studies of ocular surface disorders for the potential identification of unique biomarkers for diagnostic and therapeutic applications. In this paper we identified a number of potential biomarkers of OA, with the most interesting being KRT76, FMOD and CAPZA 1/2. Future studies repeated in greater populations will be used to confirm and address these findings, with potential to provide much needed insight into the biological processes affected by OA, and thus, how allergy-induced itch and eye-rubbing impact the ocular surface.

## Data availability statement

The datasets presented in this study can be found in online repositories. The names of the repository/repositories and accession number(s) can be found below: PXD051204 (PRIDE).

## Ethics statement

The studies involving humans were approved by the Deakin University Human Research Ethics Committee (DUHREC study #2021-189). The studies were conducted in accordance with the local legislation and institutional requirements. The participants provided their written informed consent to participate in this study.

## Author contributions

EA: Conceptualization, Data curation, Formal analysis, Investigation, Methodology, Visualization, Writing – original draft, Writing – review & editing. SN: Conceptualization, Data curation, Methodology, Writing – original draft, Writing – review & editing. SA: Conceptualization, Supervision, Writing – original draft, Writing – review & editing. LC: Conceptualization, Supervision, Writing – original draft, Writing – review & editing. MG: Conceptualization, Supervision, Writing – original draft, Writing – review & editing. CS: Conceptualization, Funding acquisition, Project administration, Supervision, Writing – review & editing.
