# Supplementary material for: What’s the situation with ocular inflammation? A cross-seasonal investigation of proteomic changes in ocular allergy sufferers’ tears in Victoria, Australia
Source: Front Immunol. 2024 May 24;15:1386344. doi: 10.3389/fimmu.2024.1386344 (PMC11157006; doi:10.3389/fimmu.2024.1386344)
Supplement: Supplementary file 1 [file Table_1.docx]

**Supplementary Data**

Supplementary Table 1- Differentially expressed proteins in human tears of Ocular Allergy (OA) sufferers and Healthy Controls (HC) across seasons. Significantly differentially expressed proteins (p-value<0.05) were filtered by Log_2_ fold-change.

| **Gene name** | **Protein name** | **Fold change (Log_2_)** | **p-value** |
| --- | --- | --- | --- |
| ***Upregulated in peak allergy season OA vs HC*** | | | |
| **DHX35** | Probable ATP-dependent RNA helicase DHX35 | 2.08 | 0.012 |
| **AMY1A/B/C** | Alpha-amylase 1B;Alpha-amylase 1C;Alpha-amylase 1A | 2.03 | 0.0193 |
| **KRT76** | Keratin, type II cytoskeletal 2 oral | 1.87 | 0.0498 |
| **IGLL1** | Immunoglobulin lambda-like polypeptide 1 | 1.7 | 0.0399 |
| **IGLV3-21** | Immunoglobulin lambda variable 3-21 | 1.45 | 0.0439 |
| **CSMD1** | CUB and sushi domain-containing protein 1 | 1.26 | 0.013 |
| **FMOD** | Fibromodulin | 1.22 | 0.0136 |
| **CTSC** | Dipeptidyl peptidase 1 | 1.16 | 0.0263 |
| **DKK4** | Dickkopf-related protein 4 | 1.14 | 0.0102 |
| ***Downregulated in peak allergy season OA vs HC*** | | | |
| **DDOST** | Dolichyl-diphosphooligosaccharide--protein glycosyltransferase | -1.03 | 0.0498 |
| **CAPZA1/2** | F-actin-capping protein subunit alpha | -1.03 | 0.0431 |
| **MSN** | Moesin | -1.11 | 0.0218 |
| **SNRPA1** | Small nuclear ribonucleoprotein polypeptide A | -1.11 | 0.0159 |
| **RNASE7** | Ribonuclease 7 | -1.17 | 0.031 |
| **DUT** | Deoxyuridine 5'-triphosphate nucleotidohydrolase | -1.31 | 0.0431 |
| **UQCRB** | Cytochrome b-c1 complex subunit 7 | -1.46 | 0.0034 |
| **C9** | Complement component C9 | -1.5 | 0.0366 |
| **KNG1** | Kininogen-1 | -1.53 | 0.0453 |
| **SEPTIN2** | Septin-2 | -1.65 | 0.0321 |
| **FEN1** | Flap endonuclease 1 | -1.85 | 0.0031 |
| **ELANE** | Neutrophil elastase | -2.01 | 0.0223 |
| **CTSG** | Cathepsin G | -2.14 | 0.0416 |
| ***Upregulated in off-peak season OA vs HC*** | | | |
| **IGHG2** | Immunoglobulin heavy constant gamma 2 | 1.84 | 0.034 |
| **IGHV1-69** | Immunoglobulin heavy variable 1-69 | 1.81 | 0.026 |
| **IGLC7** | Immunoglobulin lambda constant 7 | 1.57 | 0.045 |
| **IGKV1-8** | Immunoglobulin kappa variable 1-8 | 1.47 | 0.036 |
| **ANXA3** | Annexin A3 | 1.22 | 0.021 |
| **SH3RF2** | E3 ubiquitin-protein ligase | 1.13 | 0.046 |
| **VTN** | Vitronectin | 1.09 | 0.028 |
| **ANXA11** | Annexin A11 | 1.04 | 0.025 |
| ***Downregulated in off-peak season OA vs HC*** | | | |
| **DLST** | Dihydrolipoyllysine-residue succinyltransferase component of 2-oxoglutarate dehydrogenase complex, mitochondrial | -1.01 | 0.037 |
| **TARS1** | Threonine--tRNA ligase 1, cytoplasmic | -1.03 | 0.031 |
| **TTLL12** | Tubulin--tyrosine ligase-like protein 12 | -1.05 | 0.045 |
| **RTN1** | Reticulon-1 | -1.07 | 0.021 |
| **DSC3** | Desmocollin-3 | -1.14 | 0.028 |
| **RPS10** | 40S ribosomal protein S10 | -1.16 | 0.039 |
| **SLC25A4** | ADP/ATP translocase 1 | -1.21 | 0.031 |
| **NANS** | Sialic acid synthase | -1.32 | 0.019 |
| **SSBP1** | Single-stranded DNA-binding protein, mitochondrial | -1.42 | 0.021 |
| **NOP58** | Nucleolar protein 58 | -1.52 | 0.024 |
| **G3BP1** | Ras GTPase-activating protein-binding protein 1 | -1.59 | 0.013 |
| **CAPZA1; CAPZA2** | F-actin-capping protein subunit alpha | -1.67 | 0.046 |
| **MYDGF** | Myeloid-derived growth factor | -2.36 | 0.031 |
| ***Upregulated in peak allergy season HC vs off-peak season HC*** | | | |
| **ELANE** | Neutrophil elastase | 2.57 | 0.029 |
| **IGLC7** | Immunoglobulin lambda constant 7 | 2.14 | 0.035 |
| **GRIP1** | Glutamate receptor-interacting protein 1 | 1.87 | 0.039 |
| **ANXA3** | Annexin 3 | 1.72 | 0.006 |
| ***Downregulated in peak allergy season HC vs off-peak season HC*** | | | |
| **CDSN** | Corneodesmosin | -1.09 | 0.050 |
| **KRT9** | Keratin, type I cytoskeletal 9 | -1.10 | 0.040 |
| **MAP7D1** | Arginine/proline rich coiled-coil 1, isoform CRA_b;MAP7 domain-containing protein 1 | -1.15 | 0.006 |
| **RNH1** | Ribonuclease inhibitor | -1.16 | 0.037 |
| **ALDH1A1** | Aldehyde dehydrogenase 1A1 | -1.16 | 0.036 |
| **KRT16** | Keratin, type I cytoskeletal 16 | -1.69 | 0.016 |
| **KRT6B** | Keratin, type II cytoskeletal 6B | -1.72 | 0.001 |
| **CSMD1** | CUB and sushi domain-containing protein 1 | -1.73 | 0.050 |
| **GCNT3** | Beta-1,3-galactosyl-O-glycosyl-glycoprotein beta-1,6-N-acetylglucosaminyltransferase 3 | -1.86 | 0.031 |
| ***Upregulated in peak allergy season OA vs off-peak season OA*** | | | |
| **IGKV3-7** | Probable non-functional immunoglobulin kappa variable 3-7 | 1.60 | 0.047 |
| **STK11IP** | Serine/threonine-protein kinase 11-interacting protein | 1.57 | 0.033 |
| **EEF1DP3** | Putative elongation factor 1-delta-like protein | 1.21 | 0.028 |
| ***Downregulated in peak allergy season OA vs off-peak season OA*** | | | |
| **TGM3** | TGc domain-containing protein;Protein-glutamine gamma-glutamyltransferase E | -1.05 | 0.019 |
| **IAH1** | Isoamyl acetate-hydrolyzing esterase 1 homolog | -1.28 | 0.020 |
| **BIVM** | Basic immunoglobulin-like variable motif-containing protein | -1.34 | 0.044 |
| **KRT77** | Keratin, type II cytoskeletal 1b | -1.42 | 0.005 |
| **MYO1F** | Unconventional myosin-If | -1.48 | 0.026 |
| **TRPM3** | Transient receptor potential cation channel subfamily M member 3 | -1.75 | 0.040 |
| **SCAND1** | SCAN domain-containing protein 1 | -2.04 | 0.039 |
| **KERA** | Keratocan | -2.53 | 0.007 |
